# Correction to “Porcine kidney xenotransplantation: From primate models to clinical reality”

**DOI:** 10.1002/ame2.70284

**Published:** 2026-08-18

Guo Z , Zhang L , Deng S , Qin C . Porcine kidney xenotransplantation: from primate models to clinical reality. Animal Model Exp Med. 2026;9(6):1142‐1166. doi:10.1002/ame2.70195 42104578 PMC13383918

1. There is a minor error in Figure 1A. The positions of N‐glycolylneuraminic acid (Neu5Gc) and α‐Gal should be exchanged. The corrected Figure 1A is provided below.

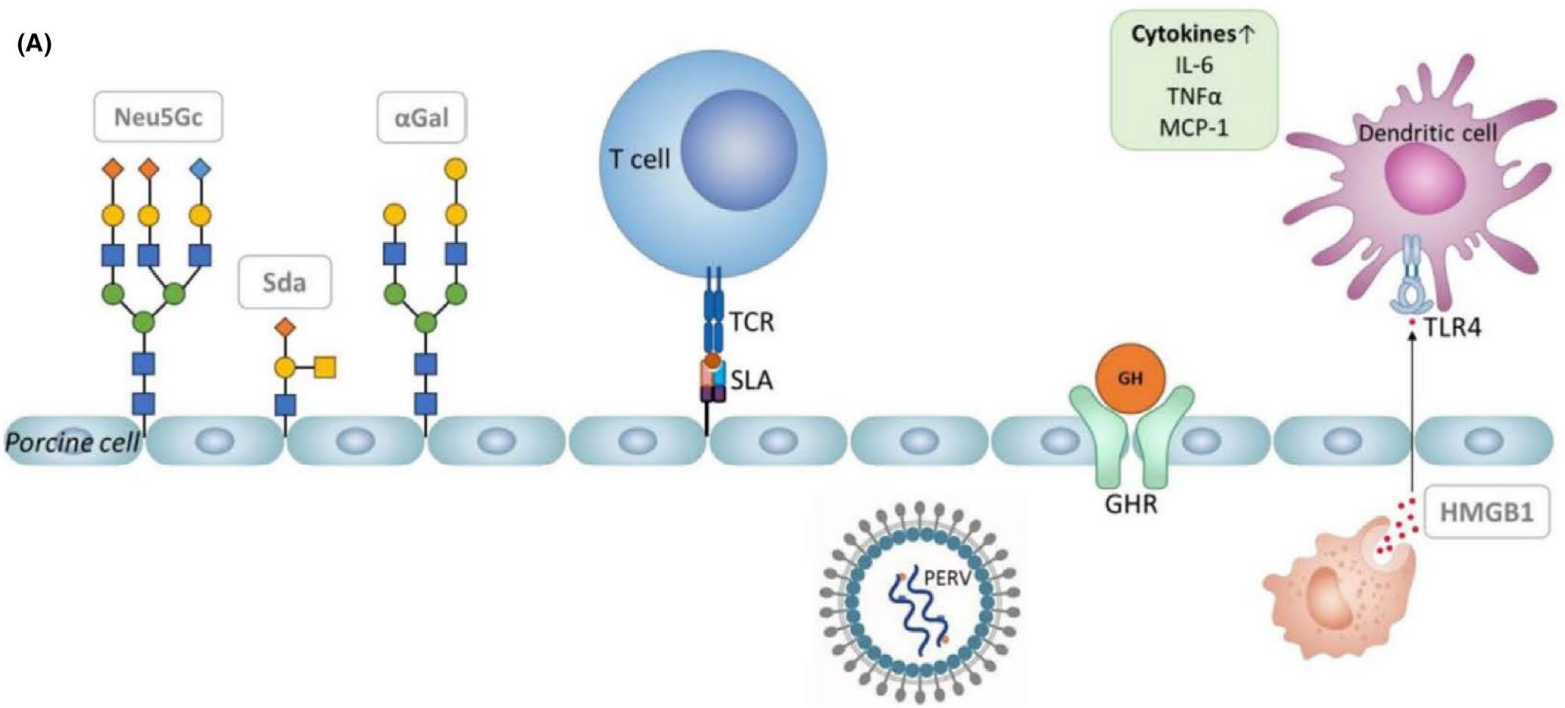

2. We would like to add the following text to the Acknowledgments section:

We thank Thomas Binder for editing this manuscript. Thomas Binder is affiliated with the Institut für Transfusionsmedizin, Universitätsmedizin Rostock (UMR).

The corrected Acknowledgments section should read as follows:

**ACKNOWLEDGMENTS**

This work was supported by the CAMS Innovation Fund for Medical Sciences (CIFMS) (2025‐I2M‐FGS‐005) and the Non‐profit Central Research Institute Fund of the Chinese Academy of Medical Sciences (2023‐PT180‐01). We thank Thomas Binder for editing this manuscript. Thomas Binder is affiliated with the Institut für Transfusionsmedizin, Universitätsmedizin Rostock (UMR).

We apologize for these errors.

